# The association between systemic inflammation markers and the prevalence of hypertension

**DOI:** 10.1186/s12872-023-03661-6

**Published:** 2023-12-14

**Authors:** Nake Jin, Lei Huang, Jun Hong, Xuechen Zhao, Jianan Hu, Shanshan Wang, Xudong Chen, Jiacheng Rong, Yingjie Lu

**Affiliations:** Department of Cardiology, Ningbo Hangzhou Bay Hospital, Ningbo, 315336 China

**Keywords:** Systemic inflammation markers, Systemic immune inflammation index, System inflammation response index, Aggregate index of systemic inflammation, Hypertension

## Abstract

**Background:**

We conducted a large-scale epidemiological analysis to investigate the associations between systemic inflammation markers and hypertension prevalence. Our aim is to identify potential biomarkers for early detection of hypertension.

**Methods:**

A cross-sectional study with 119664 individuals from the National Health and Nutrition Examination Survey was performed. We investigated the associations between three systemic inflammation markers, namely the systemic immune inflammation index (SII), system inflammation response index (SIRI), and aggregate index of systemic inflammation (AISI), and the prevalence of hypertension.

**Results:**

The prevalence rates of hypertension gradually increased with increasing logSII, logSIRI, and logAISI quartiles. In continuous analyses, each unit increase in logSII, logSIRI, and logAISI was associated with a 20.3%, 20.1%, and 23.7% increased risk of hypertension. Compared to those in the lowest quartiles, the hypertension risks for subjects in the highest logSII, logSIRI, and logAISI quartiles were 1.114-fold,1.143-fold, and 1.186-fold. The restricted cubic splines (RCS) analysis revealed a non-linear relationship between the elevation of systemic inflammation markers and hypertension prevalence. Specifically, a per standard deviation increase in any of these variables is associated with a respective 9%, 16%, and 11% increase in hypertension prevalence.

**Conclusion:**

Our cross-sectional study reveals significant positive correlations between SII, SIRI, and AISI with the prevalence of hypertension.

**Supplementary Information:**

The online version contains supplementary material available at 10.1186/s12872-023-03661-6.

## Introduction

Hypertension has a significant impact on cardiovascular outcomes, including stroke, myocardial infarction, and heart failure [1]. It remains the most potent predictor of mortality as a global risk factor for death, disability-adjusted life years, and loss of life years [2]. It is estimated that 31.1% of adults worldwide (1.39 billion) had hypertension in 2010 and has emerged as the most pressing and expensive public health concern [3]. Consequently, it is imperative and of utmost importance to investigate the risk factors and efficacious predictors of hypertension to alleviate the burden on public health.

Extensive research has shown that both human and animal studies provide evidence supporting the notion that autoimmunity, inflammation and metabolic may contribute to the development of hypertension [4–6]. White blood cells and their various subpopulations, as well as platelets, are indispensable constituents of the systemic inflammatory state. Notably, recent studies have highlighted several markers of systemic inflammation in peripheral blood cells, including the systemic immune inflammation index (SII), system inflammation response index (SIRI), and aggregate index of systemic inflammation (AISI), which are associated with both cardiovascular and non-cardiovascular disorders such as heart failure, sacroiliitis, diabetic nephropathy [7–13].

Despite the observed association between SII and hypertension, as well as blood pressure [14, 15], there is currently a lack of research investigating the correlation between SIRI, AISI and hypertension, as well as no studies that concurrently compare these three systemic inflammation markers. Therefore, we present a comprehensive epidemiological analysis to gain deeper insights into the associations between SII, SIRI and AISI with both hypertension prevalence and blood pressure levels. This study ultimate objective is to gather evidence for potential biomarkers that can assist in the early detection of hypertension.

## Materials and methods

### Study population

The databases are all obtainable from the National Health and Nutritional Examination Survey (NHANES) website, which comprises a series of population-based national surveys aimed at assessing the health and nutritional status of United States citizens. We conducted data analysis on the last 12 NHANES survey cycles (1999-March 2020 Pre-pandemic), encompassing a total of 119664 participants. After excluding participants who lacked information on systemic inflammatory markers (*n* = 24773), those with unavailable hypertension diagnosis or blood pressure (*n* = 32465), and individuals under the age of 20 (*n* = 19597), a total of 42,829 participants were included in the final analysis.

### Systemic inflammatory markers

According to the NHANES protocol, automated hematology analyzing devices were used to measure lymphocytes, monocytes, neutrophils and platelets count through complete blood count analysis. Based on the counts of peripheral blood cells, we computed three systemic inflammation markers: SII, SIRI, and AISI. SII was calculated as follows: platelets count × neutrophils count / lymphocytes count. SIRI was calculated as follows: neutrophils count × monocytes count / lymphocytes count. AISI was calculated as follows: neutrophils count × platelets count × monocytescount platelets / lymphocytes count.

### Covariates information

Covariates potentially influencing the associations between three systemic inflammation markers and both hypertension prevalence and blood pressure were incorporated into this study, including gender (male/female), age (year), race (Non-white people/White people), education level (less than high school/high school /above high school/not recorded), smoking status (no/yes/not recorded), alcohol status (no/yes/not recorded), diabetes (no/yes/not recorded), hyperlipidemia(no/yes/not recorded), pulse rate, BMI, alanine transaminase (ALT), aspartate transaminase(AST), total cholesterol(TC), triglyceride(TG), low-density lipoprotein-cholesterol (LDL-C), high-density lipoprotein-cholesterol(HDL-C), glucose(GLU), HbA1c, serum uric acid, serum creatinine and C-reactive protein(CRP). Hypertension was defined as the response to the question: “Have you ever been told by a doctor or other health professional that you had hypertension, also called high blood pressure?”, and the responses were classified into three groups: yes, no and not recorded. In this study, blood pressure was assessed by measuring systolic blood pressure (SBP), diastolic blood pressure (DBP) and mean arterial pressure (MAP). The SBP and DBP were calculated as the means of all available measurement data. The MAP is calculated as (2 × DBP + SBP)/3.

### Statistical analysis

The baseline characteristics of the participants were stratified into non-hypertension and hypertension groups. Median (interquartile range) was used for continuous variables, while categorical variables were expressed in numbers and percentages. Statistical comparisons between the two groups were performed using χ^2^ tests for categorical variables, one-way ANOVA tests for normally distributed data, or Kruskal–Wallis tests for non-normally distributed data. The three systemic inflammation markers were analyzed as continuous independent variables and scaled per 1-unit increment in log-transformed or divided into quartiles to investigate their associations with hypertension prevalence. Multivariate logistic regression models were employed with various adjustments to estimate the odds ratios (ORs) and their corresponding 95% confidence intervals (CIs). To investigate the non-linear correlation between the three systemic inflammation markers and hypertension, we employ restricted cubic splines (RCS) analysis. In cases where the RCS analysis revealed a U-shaped, Inverted U-shaped, or L-shaped curve, with a clearly identifiable inflection point, the data were divided into two distinct segments based on this inflection point, and segmented regression analysis was conducted separately for each group. Furthermore, Spearman correlation analysis and one-way analysis of variance (One-way ANOVA) were utilized to investigate the associations between the three systemic inflammation markers and blood pressure. A value of *p* < 0.05 (two-sided) was considered statistically significant. All the analyses were performed with R and SPSS software.

## Results

### Baseline characteristics

The demographic characteristics of the study participants are presented in Table 1, with a total of 42,829 individuals aged between 20 and 85 years included in the analysis. There were 34.37% of the participants with hypertension, 48.44% were males, 45.91% were white peoples, and the median age was 49 years old. In general, baseline characteristics differed significantly between non-hypertension and hypertension groups, except for gender.The levels of SII, SIRI and AISI were significantly elevated in hypertensive patients compared to those without hypertension. Non-normally distributed continuous variables underwent logarithmic transformations for analysis purposes. The results showed that the difference in log-transformed SII, SIRI, and AISI remained significant.

**Table 1:** Baseline characteristics of NHANES participants included in this study

	Total (*N* = 42829)	Non-hypertension (*N* = 28109)	Hypertension (*N* = 14720)	*P*
Ages(years)	49.00(34.00,64.00)	41.00(30.00,56.00)	62(50.00,72.00)	< 0.001
Gender				0.55
Male(%)	20476(48.44)	13710(48.77)	7036(87.80)	
Female(%)	22083(51.56)	14399(51.23)	7684(52.20)	
Race				< 0.001
Non-white people(%)	23166(54.09)	15404(54.80)	7762(52.73)	
White people(%)	19663(45.91)	12705(45.20)	6958(47.27)	
Education				< 0.001
Less than high school(%)	11855(27.8)	7620(25.83)	4595(31.22)	
High school(%)	9856(23.01)	6282(22.35)	3574(24.28)	
Above high school (%)	21066(49.19)	14533(51.70)	6533(44.38)	
Not recorded(%)	52(0.12)	34(0.12)	18(0.12)	
Smoking				< 0.001
No(%)	23046(53.81)	15765(56.09)	7281(49.46)	
Yes(%)	19746(46.10)	12319(43.83)	7427(50.46)	
Not recorded(%)	37(0.09)	25(0.09)	12(0.08)	
Alcohol				< 0.001
No(%)	7740(18.07)	4098(14.58)	3642(24.74)	
Yes(%)	26361(61.55)	18380(65.39)	7980(54.22)	
Not recorded(%)	8728(20.38)	5631(20.03)	3097(21.04)	
Diabetes				< 0.001
No(%)	36963(86.30)	26172(93.11)	10791(73.31)	
Yes(%)	5011(11.70)	1595(5.67)	3416(23.21)	
Not recorded(%)	855(2.00)	342(1.22)	513(3.48)	
Hyperlipidemia				< 0.001
No(%)	20690(48.31)	14784(52.60)	5906(40.12)	
Yes(%)	13277(31.00)	5793(20.61)	7484(50.84)	
Not recorded(%)	8862(20.69)	7532(26.79)	1330(9.04)	
Pulse rate(bpm)	72.00(64.00,80.00)	72.00(64.00,80.00)	72.00(64.00,80.00)	< 0.001
BMI(kg/m^2^)	27.80(24.21,32.13)	26.85(23.55,30.90)	29.64(26.00,34.30)	< 0.001
Lymphocyte number	2.00(1.60,2.50)	2.00(1.70,2.50)	2.00(1.60,2.50)	< 0.001
Monocyte number	0.40(0.50,0.70)	0.50(0.40,0.60)	0.50(0.40,0.70)	< 0.001
Neutrophils number	4.00(3.10,5.20)	4.00(3.10,5.20)	4.10(3.20,5.20)	< 0.001
Platelet count	246.00(208.00,290.00)	247.00(210.00,291.00)	241(202.00,287.00)	< 0.001
ALT	21.00(16.00,28.00)	21.00(16.00,28.47)	21.00(17.00,28.00)	< 0.001
AST	23.00(19.00,28.00)	23.00(19.00,27.00)	23.00(20.00,28.00)	< 0.001
TC(mmol/L)	4.99(4.32,5.74)	5.02(4.34,5.72)	4.99(4.27,5.74)	0.002
TG (mmol/L)	1.37(0.90,2.09)	1.28(0.86,1.98)	1.54(1.04,2.29)	< 0.001
LDL-C (mmol/L)	2.86(2.28,3.49)	2.89(2.33,3.50)	2.82(2.20,3.47)	< 0.001
HDL-C (mmol/L)	1.29(1.08,1.60)	1.32(1.09,1.60)	1.27(1.06,1.58)	< 0.001
GLU	5.11(4.72,5.72)	5.00(4.61,5.50)	5.44(4.91,6.38)	< 0.001
HbA1c	5.50(5.20,5.80)	5.40(5.10,5.60)	5.70(5.40,6.20)	< 0.001
Serum uric acid (mmol/L)	315.20(261.70,374.70)	303.30(249.80,362.80)	339.00(285.50,404.50)	< 0.001
Serum creatinine (mmol/L)	73.37(61.88,88.40)	70.72(61.88,85.75)	79.56(66.07,97.24)	< 0.001
C-Reactive protein	0.36(0.10,1.27)	0.30(0.08,1.10)	0.50(0.15,1.62)	< 0.001
SBP(mmHg)	121(111,135)	117(109,127)	132(120,147)	< 0.001
DBP(mmHg)	71(63,78)	70(63,77)	72(63,81)	< 0.001
MAP(mmHg)	87.33(80.22,95.33)	85.56(78.89,92.44)	91.78(83.78,100.44)	< 0.001
SII	486.46(345.43,688.00)	481.25(344.51,678.33)	495.58(347.15,706.64)	< 0.001
SIRI	1.05(0.71,1.54)	1.00(0.69,1.47)	1.12(0.75,1.67)	< 0.001
AISI	255.30(165.00,396.57)	249.23(162.03,384.81)	268.80(171.60,419.98)	< 0.001
LogSII	2.69(2.54,2.84)	2.68(2.54,2.83)	2.70(2.54,2.85)	< 0.001
LogSIRI	0.02(-0.15,0.19)	0.00(-0.16,0.17)	0.05(-0.12,0.22)	< 0.001
LogAISI	2.41(2.22,2.60)	2.40(2.21,2.59)	2.43(2.23,2.62)	< 0.001

*ALT* Alanine transaminase, *AST* Aspartate transaminase, *TC* Total cholesterol, *TG* Triglyceride, *LDL-C* Low density lipoprotein cholesterol, *HDL-C* High density lipoprotein cholesterol, *GLU glucose* HbA1c glycated hemoglobin, *CRP* C-reactive protein, *SBP* systolic blood pressure, *DBP* Diastolic blood pressure, *MAP* Mean arterial pressure, *SII* Systemic immune inflammation index, *SIRI* System inflammation response index, *AISI* Aggregate index of systemic inflammation

### Systemic inflammation markers and hypertension prevalence

**Fig. 1:**
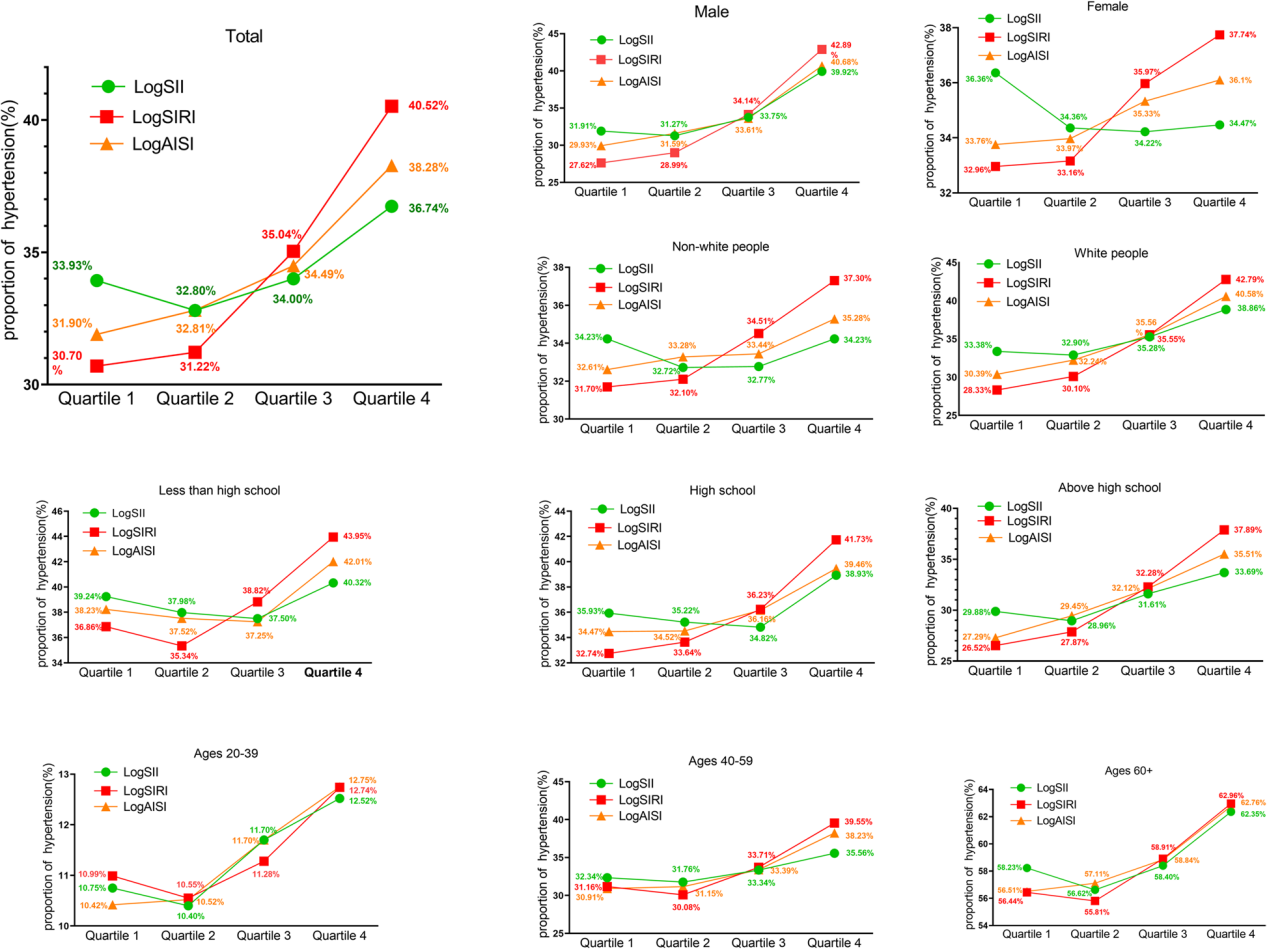
Distribution of hypertension proportions among different quartiles of the three systemic inflammation markers

To investigate the association between systemic inflammation markers and the prevalence of hypertension, we conducted further analyses by dividing subjects into four quartiles based on their log-transformed levels (Refer to Supplementary Table 1 for a detailed breakdown of the grouping). Our study investigated the prevalence rates of hypertension across quartiles of logSII, logSIRI and logAISI. For logSII, the number of patients with hypertension in quartiles 1–4 was 3633, 3513, 3638, and 3936, respectively. Prevalence rates increased from quartile 1 to quartile 4 (33.93%, 32.80%, 34.00%, and 36.74%, respectively). In logSIRI, the number of patients with hypertension in quartiles 1–4 were 3281, 3350, 3750, and 4339, respectively, with prevalence rates of 30.70%, 31.22%, 35.04%, and 40.52%. Similarly, in logAISI, the number of patients with hypertension in quartiles 1–4 were 3419, 3510, 3692, and 4099, respectively, with prevalence rates of 31.90%, 32.81%, 34.49%, and 38.28%. Overall, these results demonstrate a gradual escalation in the prevalence rates of hypertension as logSII, logSIRI, and logAISI quartiles increase (Fig. 1).

We conducted subgroup analyses stratified by participant characteristics to investigate the associations between three systemic inflammation markers and hypertension prevalence in a more detailed manner. Obviously, we have observed a gradual increase in the proportion of hypertensive patients as quartiles of logSII, logSIRI and logAISI rise within each subgroup. However, it should be noted that this trend is not applicable to the female logSII quartile (Fig. 1).

### Systemic inflammation markers and hypertension risk

**Fig. 2:**
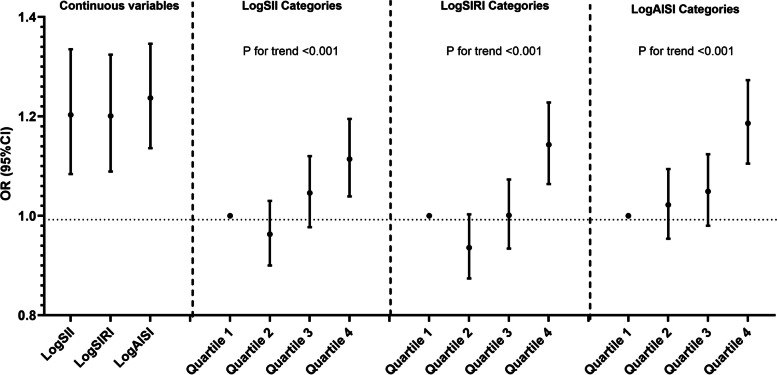
Multivariate-adjusted OR (95% CI) of the relationships between the three systemic inflammation markers and hypertension prevalence in continuous and quartiles analyses. Adjusted for gender, age, race, education, smoking, alcohol, diabetes, hyperlipidemia, pulse rate, body mass index, alanine transaminase, aspartate transaminase, total cholesterol, triglyceride, low density lipoprotein cholesterol, high density lipoprotein cholesterol, glucose, glycated hemoglobin, serum uric acid, serum creatinine and C-reactive protein

The results of the multivariate logistic regression analysis demonstrate that elevated levels of three systemic inflammation markers are associated with an increased risk of hypertension (Table 2 and Fig. 2). These associations remain significant in both the unadjusted model (Model1: logSII[OR = 1.204;95% CI,1.107–1.309, *p* < 0.001]; logSIRI[OR = 1.827;95%CI,1.694–1.972, *p* < 0.001];logAISI[OR = 1.415;95%CI, 1.323–1.531,*p* < 0.001]) and the partially adjusted model(Model2:logSII[OR = 1.204;95%CI,1.107–1.309*,p* < 0.001]; logSIRI[OR = 1.827;95%CI,1.694–1.972,*p* < 0.001];logAISI[OR = 1.415;95% CI, 1.323–1.531,*p* < 0.001]). The fully adjusted model estimated the odds ratios (ORs) for each unit increment of logSII, logSIRI and logAISI at 1.203 (95% CI: 1.084–1.335), 1.201 (95% CI: 1.089–1.324), and 1.237 (95% CI: 1.136–1.346), respectively, indicating that an increase in one unit of logSII, logSIRI and logAISI scores was associated with a respective increased risk of hypertension by 20.3%, 20.l% and 23 0.7% (Fig. 2).

**Table 2:** Associations between three systemic inflammation markers and hypertension risk

	Model 1		Model 2		Model 3	
	OR(95%CI)	*P*	OR(95%CI)	*P*	OR(95%CI)	*P*
LogSII	1.204(1.107,1.309)	< 0.001	1.310(1.189,1.444)	< 0.001	1.203(1.084,1.335)	< 0.001
LogSII categories						
Quartile 1	Reference		Reference		Reference	
Quartile 2	0.950(0.898,1.006)	0.008	0.967(0.906,1.033)	0.320	0.963(0.900,1.030)	0.273
Quartile 3	1.003(0.948,1.061)	0.923	1.071(1.003,1.143)	0.039	1.046(0.977,1.120)	0.193
Quartile 4	1.131(1.069,1.196)	< 0.001	1.189(1.114,1.270)	< 0.001	1.114(1.039,1.195)	0.002
P for trend	< 0.001		< 0.001		< 0.001	
LogSIRI	1.827(1.694,1.972)	< 0.001	1.463(1.337,1.602)	< 0.001	1.201(1.089,1.324)	< 0.001
LogSIRI categories						
Quartile 1	Reference		Reference		Reference	
Quartile 2	1.025(0.967,1.086)	0.410	0.977(0.915,1.044)	0.490	0.936(0.874,1.003)	0.059
Quartile 3	1.218(1.150,1.289)	< 0.001	1.106(1.035,1.182)	0.003	1.001(0.934,1.073)	0.977
Quartile 4	1.538(1.454,1.627)	< 0.001	1.313(1.228,1.405)	< 0.001	1.143(1.064,1.228)	< 0.001
P for trend	< 0.001		< 0.001		< 0.001	
LogAISI	1.415(1.323,1.531)	< 0.001	1.44(1.331,1.558)	< 0.001	1.237(1.136,1.346)	< 0.001
LogAISI categories						
Quartile 1	Reference		Reference		Reference	
Quartile 2	1.042(0.984,1.104)	0.156	1.053(0.986,1.124)	0.124	1.022(0.954,1.094)	0.538
Quartile 3	1.124(1.062,1.190)	< 0.001	1.135(1.063,1.212)	< 0.001	1.049(0.980,1.124)	0.169
Quartile 4	1.324(1.252,1.401)	< 0.001	1.340(1.254,1.432)	< 0.001	1.186(1.105,1.273)	< 0.001
P for trend	< 0.001		< 0.001		< 0.001	

Model 1 was not adjusted for any confounders

Model 2 was adjusted for gender, age, race,education,smoking,alcohol,diabetes and hyperlipidemia

Model 3 was adjusted for gender, age, race, education, smoking, alcohol,diabetes, hyperlipidemia, pulse rate, body mass index, alanine transaminase, aspartate transaminase, total cholesterol, triglyceride, low density lipoprotein cholesterol, high density lipoprotein cholesterol, glucose, glycated hemoglobin, serum uric acid,serum creatinine and C-reactive protein

*OR* Odds Ratio, *CI* Confidence Interval

To perform a sensitivity analysis, we stratified logSII, logSIRI and logAISI into quartiles from their original continuous form. The associations of logSII, logSIRI and logAISI for hypertension risks were consistent with the trends in the continuous analyses after adjusting different models. From the fully adjusted model, compared to those in the lowest quartiles, individuals in the highest logSII, logSIRI and logAISI quartiles exhibited a 1.114-fold (OR = 1.114;95%CI, 1.039–1.195, *p* = 0.002), 1.143-fold (OR = 1.143;95%CI, 1.064–1.228, *p* < 0.001) and 1.186-fold (OR = 1.186;95%CI, 1.105–1.273, *p* < 0.001) increased prevalence of hypertension, respectively (Fig. 2).

**Fig. 3:**
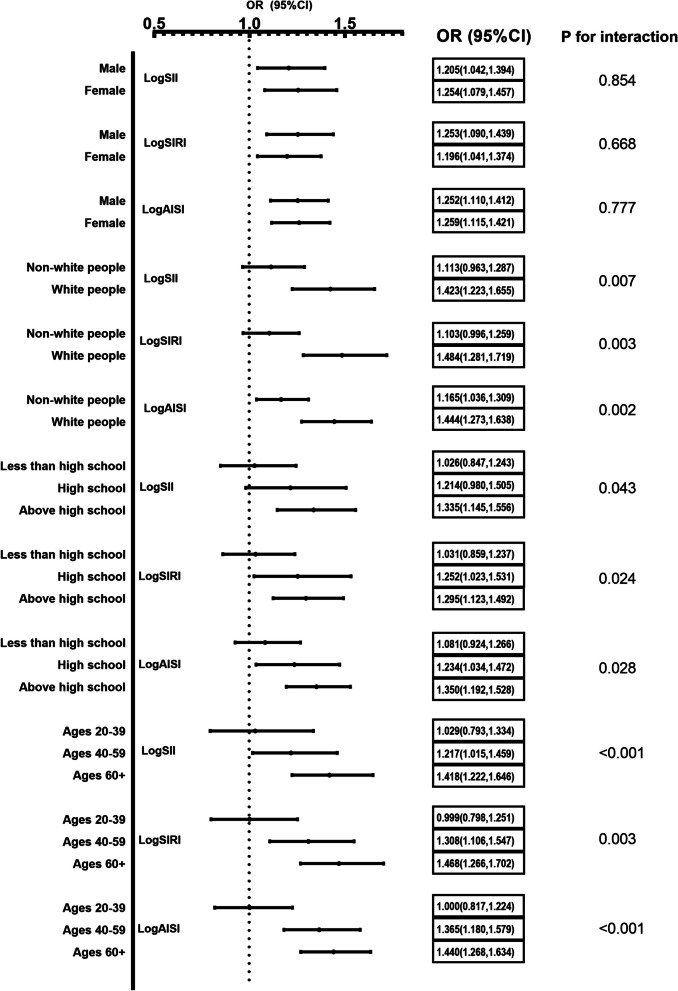
Subgroup analyses for the associations between systemic inflammation markers and the prevalence of hypertension stratified by participant characteristics. Results are expressed as multivariable-adjusted OR in continuous analyses after controlling covariates including gender, age, race, education, smoking, alcohol, diabetes, hyperlipidemia, pulse rate, body mass index, alanine transaminase, aspartate transaminase, total cholesterol, triglyceride, low density lipoprotein cholesterol, high density lipoprotein cholesterol, glucose, glycated hemoglobin, serum uric acid, serum creatinine and C-reactive protein

The subgroups were categorized by gender, age, ethnicity, and education to conduct multivariate logistic regression analysis. The associations between logSII, logSIRI and logAISI and hypertension risks were generally significant across all subgroups (Supplementary Table 2). As shown in the forest plots (Fig. 3), for logSII and logAISI, the stronger of positive associations was found among individuals who were female, older, white people and above high school. Comparatively, for logSIRI, the positive associations were found to be stronger among individuals who were male, older, white people and above high school.

**Fig. 4:**
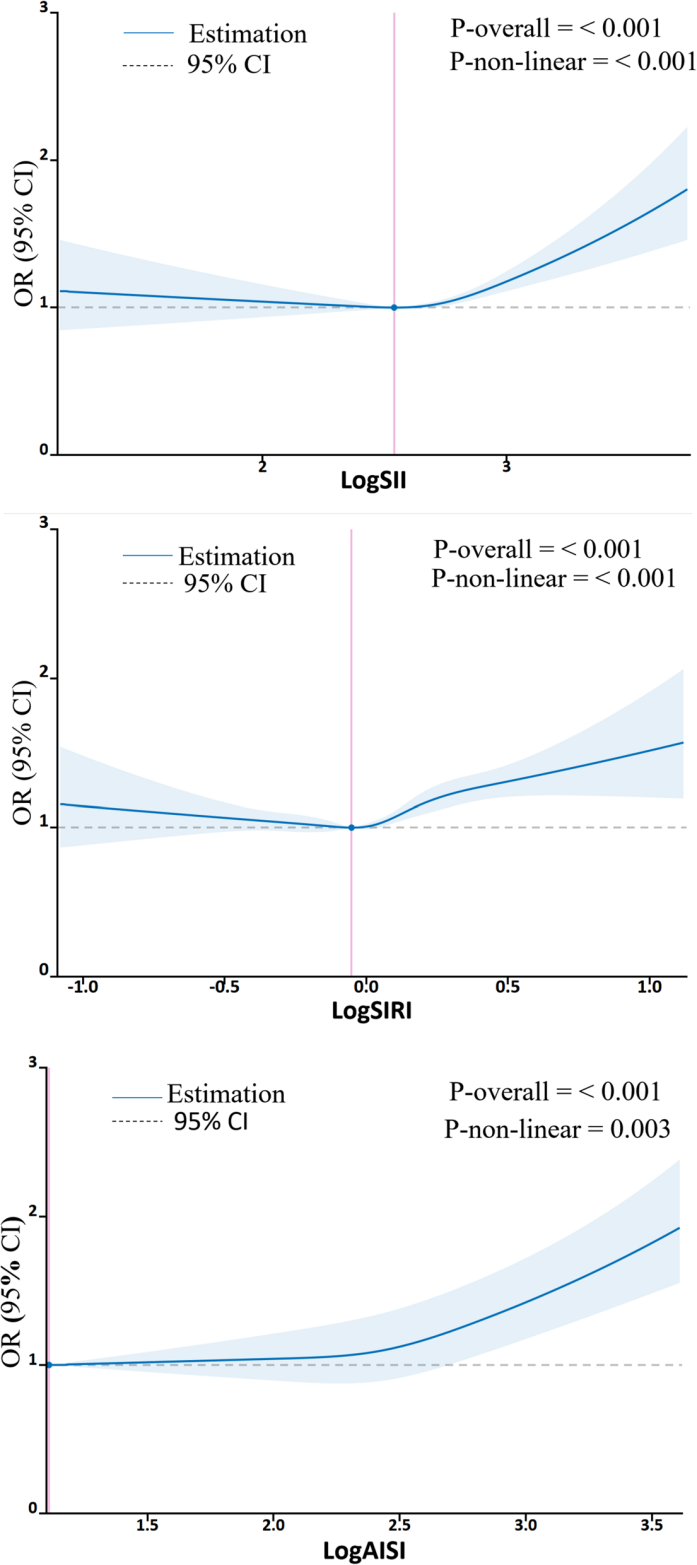
Association between systemic inflammation markers and hypertension with the RCS function. The Y-axis shows the odds ratio of having hypertension for any value of logSII, logSIRI and logAISI compared to individuals with 2.54 of logSII, -0.05 of logSIRI and 1.11 of logAISI, respectively. The logistic regression was adjusted for gender, age, race, education, smoking, alcohol, diabetes, hyperlipidemia, Pulse rate, body mass index, alanine transaminase, aspartate transaminase, total cholesterol, triglyceride, low density lipoprotein cholesterol, high density lipoprotein cholesterol, glucose, glycated hemoglobin, serum uric acid, serum creatinine and C-reactive protein

The RCS analysis found a U-shaped relationship between logSII, logSIRI, and logAISI and hypertension after adjusting for various factors. The inflection point was identified at logSII = 2.54, logSIRI = -0.05, and logAISI = 1.11, respectively (Fig. 4). By utilizing the inflection point, the data was stratified into two distinct groups. Subsequently, segmented regression analysis was conducted on each group separately. When logSII is greater than or equal to 2.54, logSIRI is greater than or equal to -0.05, and logAISI is greater than or equal to 1.11, per standard deviation increase in any of these variables is significantly associated with a respective prevalence increase of hypertension by 9%(OR = 1.09;95%CI,1.07–1.12), 16%(OR = 1.16;95%CI,1.13, 1.19)and 11%(OR = 1.11;95%CI,1.09, 1.13. The results of two piecewise linear regression models are demonstrated in Supplementary Table 3.

### Systemic inflammation markers and blood pressure

**Fig. 5:**
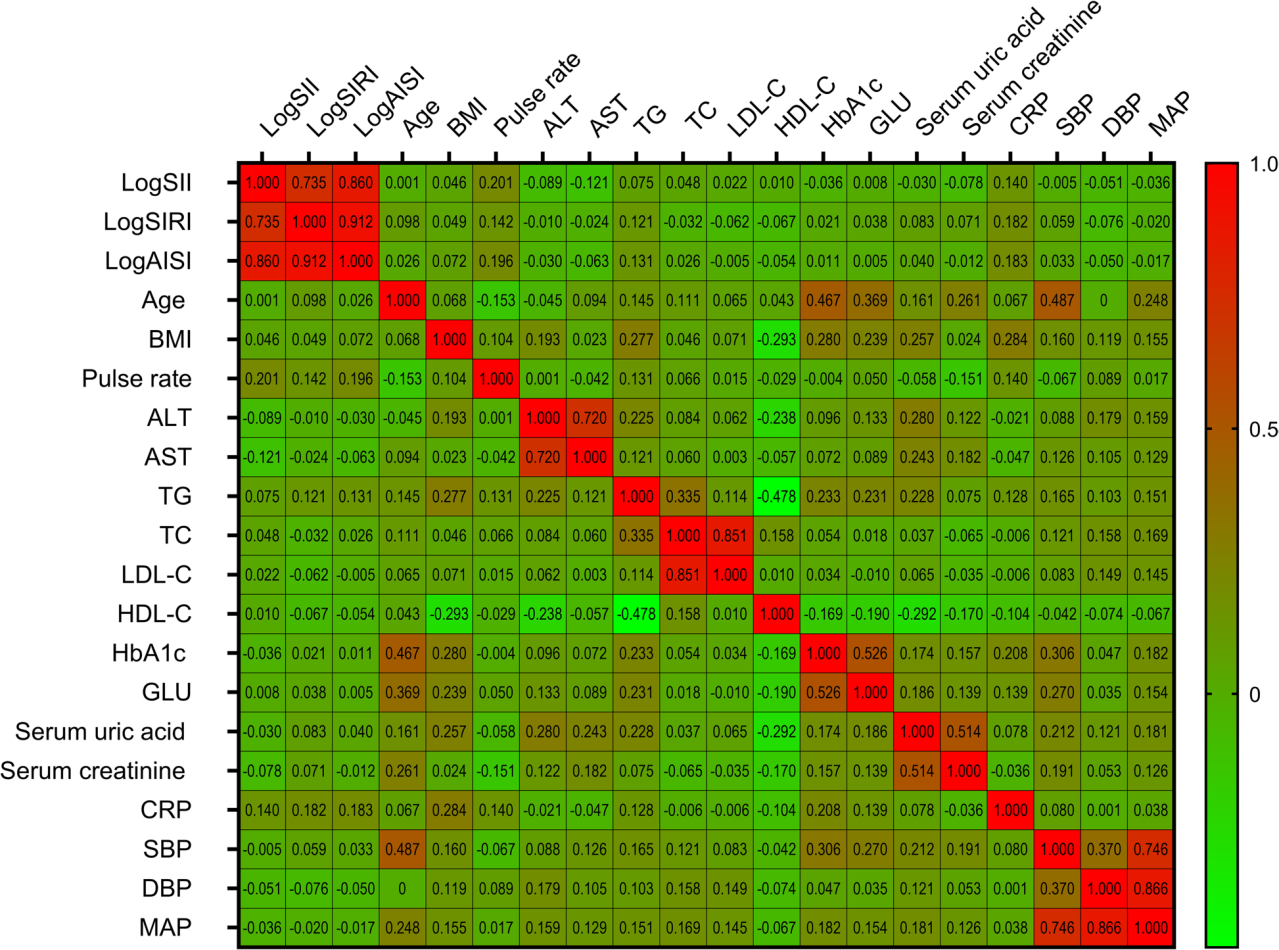
The heatmap of the correlation between covariates and blood pressure using the spearman correlation analysis among participants. ALT alanine transaminase, AST aspartate transaminase, TC total cholesterol, TG triglyceride, LDL-C low density lipoprotein cholesterol, HDL-C high density lipoprotein cholesterol, GLU glucose, HbA1c Glycated hemoglobin, CRP C-reactive protein, SBP systolic blood pressure, DBP diastolic blood pressure, MAP mean arterial pressure

**Fig. 6:**
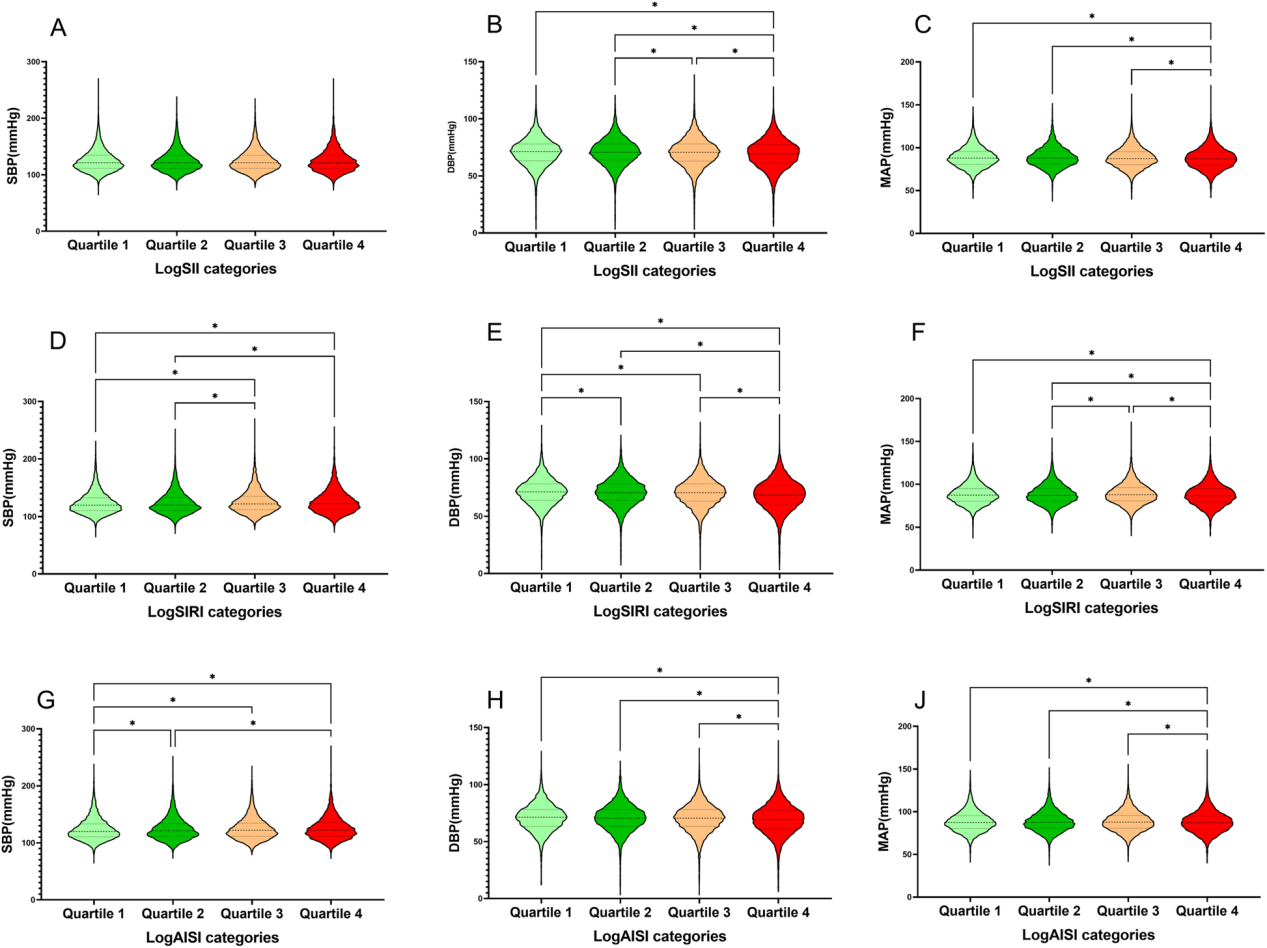
The violin plot of the correlation between blood pressure and logSII, logSIRI and logAISI quartiles. SBP systolic blood pressure, DBP diastolic blood pressure, MAP mean arterial pressure

In addition, Spearman correlation analyses and One-way ANOVA were conducted to evaluate the associations between systemic inflammation markers and blood pressure. As shown in Fig. 5 and Supplementary Table 4, inverted correlations were observed between logSII and SBP/DBP, whereas positive and inverse associations were found between logSIRI and logAISI for systolic and diastolic blood pressure, respectively. There was a negative correlation observed between MAP and logSII, logSIRI, as well as logAISI. The violin plot clearly illustrates the relationship between blood pressure and systemic inflammation markers, indicating a significant difference in blood pressure among quartile groups, with the exception of systolic blood pressure in the logSII quartile group (Fig. 6).

## Discussion

In this study, we conducted a comprehensive evaluation of different systemic inflammation markers in association with the prevalence of hypertension. The major findings of our study were as follows: (1) The levels of SII, SIRI and AISI were significantly elevated in hypertensive patients compared to those without hypertension. Furthermore, the prevalence rates of hypertension gradually increased with increasing logSII, logSIRI, and logAISI quartiles. (2) Logistic regression analysis indicated that elevated levels of three systemic inflammation markers are associated with an increased risk of hypertension. (3) The RCS analysis revealed that when logSII is ≥ 2.54, logSIRI is ≥ -0.05, and logAISI is ≥ 1.11, hypertension prevalence increases with the elevation of these systemic inflammation markers in a non-linear relationship. (4) LogSII showed an inverse correlation with SBP/DBP, while logSIRI exhibited positive and negative associations with SBP and DBP, respectively. Additionally, there was a negative correlation between MAP and logSII, logSIRI, as well as logAISI.

This is the first study to evaluate the association between SII, SIRI and AISI with hypertension prevalence using a large sample size. One of the major findings of our study is consistent with previous research conducted by Xu et al. [14], which is that SII serves as a significant predictor for hypertension prevalence. In contrast to those studies, we have additionally employed RCS analysis to explore the non-linear correlation between SII and hypertension prevalence. It is worth noting that Xu et al. does not conduct RCS analysis to assess the correlation between SII and hypertension prevalence. Moreover, limited studies with smaller sample sizes have provided supportive evidence for the correlation between SII and hypertension prevalence [16, 17], while no studies have investigated the correlation between SIRI and AISI and hypertension prevalence. Inanc et al. found that the median SII was significantly higher in the newly diagnosed hypertension group compared to the control group [16]. A further discovery showed that SIRI may be the most one of the three effective systemic inflammation markers for identifying hypertension, with the highest odds ratios and broadest applicability across different subgroups and sensitivity analyses.

As emerging biomarkers, the superiority of SII, SIRI and AISI have been identified in numerous diseases, including cancers, cardiovascular diseases, sacroiliitis and diabetic nephropathy [9, 12, 13, 18–20]. Compared to traditional markers of inflammation, the three systemic inflammation markers are more favorable indicators of inflammatory status and have demonstrated superior predictive power and prognostic value in multiple studies [21–24]. According to Yang's research, the predictive capacity of SII for major cardiovascular events in patients with coronary artery disease (CAD) who have undergone coronary intervention exceeds that of traditional risk factors [19]. Consistently, another study reported that SII and SIRI are strongly linked to cardiovascular and all-cause mortality, highlighting the importance of addressing systemic inflammation for better prevention strategies [25]. Also, increasing tertiles of AISI and SIRI significantly raised the risk of major adverse cardiovascular events (MACE) in patients with acute coronary syndrome undergoing percutaneous coronary intervention [9]. The patients exhibiting a heightened inflammatory response should be managed with more aggressive treatment strategies to mitigate the occurrence of adverse events [26]. Telemedicine and mHealth systems can be employed in such patient populations to attenuate the inflammatory response [27, 28].

Despite numerous studies indicating inflammatory factors and their resultant products, such as IL-1β, IL-6, IFN-γ, TNF-α and CRP, have been demonstrated to contribute to the pathogenesis of hypertension [29–31], there remains a paucity of research examining the association between these novel systemic inflammatory markers and hypertension. A study has demonstrated that the SII level is significantly higher in non-dipper hypertensive patients compared to dipper hypertensive patients, and SII was an independent predictor of non-dipper hypertensive [32]. Furthermore, SII is positively associated with hypertension prevalence in cross-sectional study, indicating that SII may be a superior systemic inflammation marker for predicting hypertension [14]. In addition, our study represents the first attempt to investigate the correlation between SIRI and AISI and hypertension prevalence. Inflammation contributes to hypertension by causing oxidative stress, impairing endothelial function, and promoting vascular remodeling [33]. The presence of hypertension is correlated with increased levels of metabolic and inflammatory biomarkers, thus prompting the use of combination antihypertensive therapy as a viable strategy for mitigating inflammation [6, 34, 35]. Thus, these new systemic inflammatory markers, as well as traditional inflammation factors, may provide a simple and reliable method for assessing hypertension risk in individuals with varying degrees of inflammatory status. On the other hand, our data revealed that there were significant and conflicting associations between logSII, logSIRI and logAISI with blood pressure. Therefore, inflammation may only be one of many factors contributing to hypertension risk evaluation as it is a long-term result of multiple factors such as genetics, hormones, vascular abnormalities and environmental interference [36].

There are certain inherent limitations in our study. Firstly, due to the cross-sectional nature of our research, we cannot establish any causal associations between these markers and hypertension prevalence. Furthermore, due to the unavailability of follow-up peripheral blood cell counts, we were unable to evaluate the influence of individual inflammation ratios on hypertension prevalence. Finally, despite our best efforts to adjust for potential confounding factors, there may still be some that have influenced our results. Therefore, caution should be exercised when interpreting these findings in clinical practice.

In conclusion, the prevalence rates of hypertension gradually increased with increasing logSII, logSIRI, and logAISI quartiles. Each unit increase in logSII, logSIRI, and logAISI was associated with a 20.3%, 20.1%, and 23.7% increased risk of hypertension. Compared to those in the lowest quartiles, the hypertension risks for subjects in the highest logSII, logSIRI, and logAISI quartiles were 1.114-fold,1.143-fold, and 1.186-fold. The RCS analysis revealed a non-linear relationship between the elevation of systemic inflammation markers and hypertension prevalence, with logSII ≥ 2.54, logSIRI ≥ -0.05, and logAISI ≥ 1.11 being significant predictors. Specifically, a per standard deviation increase in any of these variables is associated with a respective 9%, 16%, and 11% increase in hypertension prevalence.Our study suggests that the new systemic inflammatory markers, combined with traditional inflammation factors, can provide a simple and reliable method to assess hypertension risk in individuals with varying levels of inflammation.

### Supplementary Information

**Additional file 1: Supplementary Table 1.** Systemic inflammation markers and hypertension prevalence.

**Additional file 2: Supplementary Table 2.** Subgroup analyses for the associations between four systemic inflammation markers and hypertension risk stratified by participant characteristics in continuous analyses.

**Additional file 3: Supplementary Table 3.** Effect of standardized systemic inflammation markers on hypertension: adjusted odds ratios from segmented logistic regression analysis.

**Additional file 4: Supplementary Table 4.** Spearman correlation analysis between baseline variables and blood pressure.

## Data Availability

The datasets generated and/or analysed during the current study are available from the U.S. Department of Health and Human Services, Centers for Disease Control and Prevention [https://wwwn.cdc.gov/nchs/nhanes].
