# Supplementary material for: The association between systemic inflammation markers and the prevalence of hypertension
Source: BMC Cardiovasc Disord. 2023 Dec 14;23:615. doi: 10.1186/s12872-023-03661-6 (PMC10720087; doi:10.1186/s12872-023-03661-6)
Supplement: Supplementary file 1 — Additional file 1: Supplementary Table 1. Systemic inflammation markers and hypertension prevalence. [file 12872_2023_3661_MOESM1_ESM.docx]

| Supplementary Table 1 Systemic inflammation markers and hypertension prevalence | | | | | |
| --- | --- | --- | --- | --- | --- |
|  |  | Non-hypertension  (n) | Hypertension  (n) | Hypertension  Prevalence(%) | P |
| LogSII |  |  |  |  | ＜0.001 |
|  | Quartile 1（＜2.54） | 7073 | 3633 | 33.93 |  |
|  | Quartile 2（2.54-2.69） | 7196 | 3513 | 32.80 |  |
|  | Quartile 3（2.69-2.84） | 7063 | 3638 | 34.00 |  |
|  | Quartile 4（＞2.84） | 6777 | 3936 | 36.74 |  |
| LogSIRI |  |  |  |  | ＜0.001 |
|  | Quartile 1（＜-0.15） | 7407 | 3281 | 30.70 |  |
|  | Quartile 2（-0.15-0.02） | 7381 | 3350 | 31.22 |  |
|  | Quartile 3（0.02-0.19） | 6952 | 3750 | 35.04 |  |
|  | Quartile 4（＞0.19） | 6369 | 4339 | 40.52 |  |
| LogAISI |  |  |  |  | ＜0.001 |
|  | Quartile 1（＜2.22） | 7299 | 3419 | 31.90 |  |
|  | Quartile 2（2.22-2.41） | 7189 | 3510 | 32.81 |  |
|  | Quartile 3（2.41-2.60） | 7012 | 3692 | 34.49 |  |
|  | Quartile 4（＞2.60） | 6609 | 4099 | 38.28 |  |
|  |  |  |  |  |  |
| Male | | | | | |
| LogSII |  |  |  |  | ＜0.001 |
|  | Quartile 1（＜2.54） | 3971 | 1861 | 31.91 |  |
|  | Quartile 2（2.54-2.69） | 3706 | 1686 | 31.27 |  |
|  | Quartile 3（2.69-2.84） | 3353 | 1708 | 33.75 |  |
|  | Quartile 4（＞2.84） | 2680 | 1781 | 39.92 |  |
| LogSIRI |  |  |  |  | ＜0.001 |
|  | Quartile 1（＜-0.15） | 3271 | 1248 | 27.62 |  |
|  | Quartile 2（-0.15-0.02） | 3556 | 1452 | 28.99 |  |
|  | Quartile 3（0.02-0.19） | 3579 | 1855 | 34.14 |  |
|  | Quartile 4（＞0.19） | 3304 | 2481 | 42.89 |  |
| LogAISI |  |  |  |  | ＜0.001 |
|  | Quartile 1（＜2.22） | 3649 | 1559 | 29.93 |  |
|  | Quartile 2（2.22-2.41） | 3579 | 1653 | 31.59 |  |
|  | Quartile 3（2.41-2.60） | 3464 | 1754 | 33.61 |  |
|  | Quartile 4（＞2.60） | 3018 | 2070 | 40.68 |  |
|  |  |  |  |  |  |
| Female | | | | | |
| LogSII |  |  |  |  | 0.079 |
|  | Quartile 1（＜2.54） | 3102 | 1772 | 36.36 |  |
|  | Quartile 2（2.54-2.69） | 3490 | 1827 | 34.36 |  |
|  | Quartile 3（2.69-2.84） | 3710 | 1930 | 34.22 |  |
|  | Quartile 4（＞2.84） | 4097 | 2155 | 34.47 |  |
| LogSIRI |  |  |  |  | ＜0.001 |
|  | Quartile 1（＜-0.15） | 4136 | 2033 | 32.96 |  |
|  | Quartile 2（-0.15-0.02） | 3825 | 1898 | 33.16 |  |
|  | Quartile 3（0.02-0.19） | 3373 | 1895 | 35.97 |  |
|  | Quartile 4（＞0.19） | 3065 | 1858 | 37.74 |  |
| LogAISI |  |  |  |  | 0.027 |
|  | Quartile 1（＜2.22） | 3650 | 1860 | 33.76 |  |
|  | Quartile 2（2.22-2.41） | 3610 | 1857 | 33.97 |  |
|  | Quartile 3（2.41-2.60） | 3548 | 1938 | 35.33 |  |
|  | Quartile 4（＞2.60） | 3591 | 2029 | 36.1 |  |
|  |  |  |  |  |  |
| White people | | | | | |
| LogSII |  |  |  |  | ＜0.001 |
|  | Quartile 1（＜2.54） | 2473 | 1239 | 33.38 |  |
|  | Quartile 2（2.54-2.69） | 3289 | 1613 | 32.90 |  |
|  | Quartile 3（2.69-2.84） | 3390 | 1848 | 35.28 |  |
|  | Quartile 4（＞2.84） | 3553 | 2258 | 38.86 |  |
| LogSIRI |  |  |  |  | ＜0.001 |
|  | Quartile 1（＜-0.15） | 2279 | 901 | 28.33 |  |
|  | Quartile 2（-0.15-0.02） | 3307 | 1424 | 30.10 |  |
|  | Quartile 3（0.02-0.19） | 3524 | 1944 | 35.55 |  |
|  | Quartile 4（＞0.19） | 3595 | 2689 | 42.79 |  |
| LogAISI |  |  |  |  | ＜0.001 |
|  | Quartile 1（＜2.22） | 2387 | 1042 | 30.39 |  |
|  | Quartile 2（2.22-2.41） | 3295 | 1568 | 32.24 |  |
|  | Quartile 3（2.41-2.60） | 3421 | 1888 | 35.56 |  |
|  | Quartile 4（＞2.60） | 3602 | 2460 | 40.58 |  |
|  |  |  |  |  |  |
| Non-white people | | | | | |
| LogSII |  |  |  |  | 0.124 |
|  | Quartile 1（＜2.54） | 4600 | 2394 | 34.23 |  |
|  | Quartile 2（2.54-2.69） | 3907 | 1900 | 32.72 |  |
|  | Quartile 3（2.69-2.84） | 3673 | 1790 | 32.77 |  |
|  | Quartile 4（＞2.84） | 3224 | 1678 | 34.23 |  |
| LogSIRI |  |  |  |  | ＜0.001 |
|  | Quartile 1（＜-0.15） | 5128 | 2380 | 31.70 |  |
|  | Quartile 2（-0.15-0.02） | 4074 | 1926 | 32.10 |  |
|  | Quartile 3（0.02-0.19） | 3428 |  | 34.51 |  |
|  | Quartile 4（＞0.19） | 2774 | 1650 | 37.30 |  |
| LogAISI |  |  |  |  | 0.025 |
|  | Quartile 1（＜2.22） | 4912 | 2377 | 32.61 |  |
|  | Quartile 2（2.22-2.41） | 3894 | 1942 | 33.28 |  |
|  | Quartile 3（2.41-2.60） | 3591 | 1804 | 33.44 |  |
|  | Quartile 4（＞2.60） | 3007 | 1639 | 35.28 |  |
|  |  |  |  |  |  |
| Less than high school | | | | | |
| LogSII |  |  |  |  | 0.113 |
|  | Quartile 1（＜2.54） | 1860 | 1201 | 39.24 |  |
|  | Quartile 2（2.54-2.69） | 1819 | 1114 | 37.98 |  |
|  | Quartile 3（2.69-2.84） | 1843 | 1106 | 37.50 |  |
|  | Quartile 4（＞2.84） | 1738 | 1174 | 40.32 |  |
| LogSIRI |  |  |  |  | ＜0.001 |
|  | Quartile 1（＜-0.15） | 1836 | 1072 | 36.86 |  |
|  | Quartile 2（-0.15-0.02） | 1927 | 1053 | 35.34 |  |
|  | Quartile 3（0.02-0.19） | 1819 | 1154 | 38.82 |  |
|  | Quartile 4（＞0.19） | 1678 | 1316 | 43.95 |  |
| LogAISI |  |  |  |  | ＜0.001 |
|  | Quartile 1（＜2.22） | 1840 | 1139 | 38.23 |  |
|  | Quartile 2（2.22-2.41） | 1855 | 1114 | 37.52 |  |
|  | Quartile 3（2.41-2.60） | 1838 | 1091 | 37.25 |  |
|  | Quartile 4（＞2.60） | 1727 | 1251 | 42.01 |  |
|  |  |  |  |  |  |
| High school | | | | | |
| LogSII |  |  |  |  | 0.009 |
|  | Quartile 1（＜2.54） | 1514 | 849 | 35.93 |  |
|  | Quartile 2（2.54-2.69） | 1536 | 835 | 35.22 |  |
|  | Quartile 3（2.69-2.84） | 1651 | 882 | 34.82 |  |
|  | Quartile 4（＞2.84） | 1581 | 1008 | 38.93 |  |
| LogSIRI |  |  |  |  | ＜0.001 |
|  | Quartile 1（＜-0.15） | 1549 | 754 | 32.74 |  |
|  | Quartile 2（-0.15-0.02） | 1566 | 794 | 33.64 |  |
|  | Quartile 3（0.02-0.19） | 1635 | 929 | 36.23 |  |
|  | Quartile 4（＞0.19） | 1532 | 1097 | 41.73 |  |
| LogAISI |  |  |  |  | ＜0.001 |
|  | Quartile 1（＜2.22） | 1504 | 791 | 34.47 |  |
|  | Quartile 2（2.22-2.41） | 1550 | 817 | 34.52 |  |
|  | Quartile 3（2.41-2.60） | 1617 | 916 | 36.16 |  |
|  | Quartile 4（＞2.60） | 1611 | 1050 | 39.46 |  |
|  |  |  |  |  |  |
| Above high school | | | | | |
| LogSII |  |  |  |  | ＜0.001 |
|  | Quartile 1（＜2.54） | 3693 | 1574 | 29.88 |  |
|  | Quartile 2（2.54-2.69） | 3832 | 1562 | 28.96 |  |
|  | Quartile 3（2.69-2.84） | 3561 | 1646 | 31.61 |  |
|  | Quartile 4（＞2.84） | 3447 | 1751 | 33.69 |  |
| LogSIRI |  |  |  |  | ＜0.001 |
|  | Quartile 1（＜-0.15） | 4015 | 1449 | 26.52 |  |
|  | Quartile 2（-0.15-0.02） | 3880 | 1499 | 27.87 |  |
|  | Quartile 3（0.02-0.19） | 3488 | 1663 | 32.28 |  |
|  | Quartile 4（＞0.19） | 3150 | 1922 | 37.89 |  |
| LogAISI |  |  |  |  | ＜0.001 |
|  | Quartile 1（＜2.22） | 3948 | 1482 | 27.29 |  |
|  | Quartile 2（2.22-2.41） | 3773 | 1575 | 29.45 |  |
|  | Quartile 3（2.41-2.60） | 3554 | 1682 | 32.12 |  |
|  | Quartile 4（＞2.60） | 3258 | 1794 | 35.51 |  |
|  |  |  |  |  |  |
| Not recorded | | | | | |
| LogSII |  |  |  |  | 0.083 |
|  | Quartile 1（＜2.54） | 6 | 9 | 60.00 |  |
|  | Quartile 2（2.54-2.69） | 9 | 2 | 18.18 |  |
|  | Quartile 3（2.69-2.84） | 8 | 4 | 33.33 |  |
|  | Quartile 4（＞2.84） | 11 | 3 | 21.43 |  |
| LogSIRI |  |  |  |  | 0.781 |
|  | Quartile 1（＜-0.15） | 7 | 6 | 46.15 |  |
|  | Quartile 2（-0.15-0.02） | 8 | 4 | 33.33 |  |
|  | Quartile 3（0.02-0.19） | 10 | 4 | 28.57 |  |
|  | Quartile 4（＞0.19） | 9 | 4 | 30.77 |  |
| LogAISI |  |  |  |  | 0.330 |
|  | Quartile 1（＜2.22） | 7 | 7 | 50.00 |  |
|  | Quartile 2（2.22-2.41） | 11 | 4 | 26.67 |  |
|  | Quartile 3（2.41-2.60） | 3 | 3 | 50.00 |  |
|  | Quartile 4（＞2.60） | 13 | 4（ | 23.53 |  |
|  |  |  |  |  |  |
|  |  |  |  |  |  |
| Ages 20-39 | | | | | |
| LogSII |  |  |  |  | 0.018 |
|  | Quartile 1（＜2.54） | 3170 | 382 | 10.75 |  |
|  | Quartile 2（2.54-2.69） | 3239 | 376 | 10.40 |  |
|  | Quartile 3（2.69-2.84） | 3292 | 436 | 11.70 |  |
|  | Quartile 4（＞2.84） | 3333 | 477 | 12.52 |  |
| LogSIRI |  |  |  |  | 0.022 |
|  | Quartile 1（＜-0.15） | 3466 | 428 | 10.99 |  |
|  | Quartile 2（-0.15-0.02） | 3376 | 398 | 10.55 |  |
|  | Quartile 3（0.02-0.19） | 3123 | 397 | 11.28 |  |
|  | Quartile 4（＞0.19） | 3069 | 448 | 12.74 |  |
| LogAISI |  |  |  |  | 0.004 |
|  | Quartile 1（＜2.22） | 3275 | 381 | 10.42 |  |
|  | Quartile 2（2.22-2.41） | 3231 | 380 | 10.52 |  |
|  | Quartile 3（2.41-2.60） | 3229 | 428 | 11.70 |  |
|  | Quartile 4（＞2.60） | 3299 | 482 | 12.75 |  |
|  |  |  |  |  |  |
| Ages 40-59 | | | | | |
| LogSII |  |  |  |  | 0.006 |
|  | Quartile 1（＜2.54） | 2391 | 1143 | 32.34 |  |
|  | Quartile 2（2.54-2.69） | 2415 | 1124 | 31.76 |  |
|  | Quartile 3（2.69-2.84） | 2315 | 1158 | 33.34 |  |
|  | Quartile 4（＞2.84） | 2033 | 1122 | 35.56 |  |
| LogSIRI |  |  |  |  | ＜0.001 |
|  | Quartile 1（＜-0.15） | 2673 | 1210 | 31.16 |  |
|  | Quartile 2（-0.15-0.02） | 2529 | 1088 | 30.08 |  |
|  | Quartile 3（0.02-0.19） | 2309 | 1174 | 33.71 |  |
|  | Quartile 4（＞0.19） | 1643 | 1075 | 39.55 |  |
| LogAISI |  |  |  |  | ＜0.001 |
|  | Quartile 1（＜2.22） | 2571 | 1150 | 30.91 |  |
|  | Quartile 2（2.22-2.41） | 2434 | 1101 | 31.15 |  |
|  | Quartile 3（2.41-2.60） | 2310 | 1158 | 33.39 |  |
|  | Quartile 4（＞2.60） | 1839 | 1138 | 38.23 |  |
|  |  |  |  |  |  |
| Ages 60+ | | | | | |
| LogSII |  |  |  |  | ＜0.001 |
|  | Quartile 1（＜2.54） | 1512 | 2108 | 58.23 |  |
|  | Quartile 2（2.54-2.69） | 1542 | 2013 | 56.62 |  |
|  | Quartile 3（2.69-2.84） | 1456 | 2044 | 58.40 |  |
|  | Quartile 4（＞2.84） | 1411 | 2337 | 62.35 |  |
| LogSIRI |  |  |  |  | ＜0.001 |
|  | Quartile 1（＜-0.15） | 1268 | 1643 | 56.44 |  |
|  | Quartile 2（-0.15-0.02） | 1476 | 1864 | 55.81 |  |
|  | Quartile 3（0.02-0.19） | 1520 | 2179 | 58.91 |  |
|  | Quartile 4（＞0.19） | 1657 | 2816 | 62.96 |  |
| LogAISI |  |  |  |  | ＜0.001 |
|  | Quartile 1（＜2.22） | 1453 | 1888 | 56.51 |  |
|  | Quartile 2（2.22-2.41） | 1524 | 2029 | 57.11 |  |
|  | Quartile 3（2.41-2.60） | 1473 | 2106 | 58.84 |  |
|  | Quartile 4（＞2.60） | 1471 | 2479 | 62.76 |  |
