# Supplementary material for: The association between systemic inflammation markers and the prevalence of hypertension
Source: BMC Cardiovasc Disord. 2023 Dec 14;23:615. doi: 10.1186/s12872-023-03661-6 (PMC10720087; doi:10.1186/s12872-023-03661-6)
Supplement: Supplementary file 2 — Additional file 2: Supplementary Table 2. Subgroup analyses for the associations between four systemic inflammation markers and hypertension risk stratified by participant characteristics in continuous analyses. [file 12872_2023_3661_MOESM2_ESM.docx]

| Supplementary Table 2 Subgroup analyses for the associations between four systemic inflammation markers and hypertension risk stratified by participant characteristics in continuous analyses | | | | | | | | | |
| --- | --- | --- | --- | --- | --- | --- | --- | --- | --- |
|  |  | LogSII | |  | LogSIRI | |  | LogAISI | |
|  |  | OR(95%CI) | P for interaction |  | OR(95%CI) | P for interaction |  | OR(95%CI) | P for interaction |
|  |  |  |  |  |  |  |  |  |  |
| Male |  | 1.205(1.042,1.394) | 0.854 |  | 1.253(1.090,1.439) | 0.668 |  | 1.252(1.110,1.412) | 0.777 |
| Female |  | 1.254(1.079,1.457) |  |  | 1.196(1.041,1.374) |  |  | 1.259(1.115,1.421) |  |
|  |  |  |  |  |  |  |  |  |  |
| Non-white people |  | 1.113(0.963,1.287) | 0.007 |  | 1.103(0.996,1.259) | 0.003 |  | 1.165(1.036,1.309) | 0.002 |
| White people |  | 1.423(1.223,1.655) |  |  | 1.484(1.281,1.719) |  |  | 1.444(1.273,1.638) |  |
|  |  |  |  |  |  |  |  |  |  |
| Less than high school |  | 1.026(0.847,1.243) | 0.043 |  | 1.031(0.859,1.237) | 0.024 |  | 1.081(0.924,1.266) | 0.028 |
| High school |  | 1.214(0.980,1.505) |  |  | 1.252(1.023,1.531) |  |  | 1.234(1.034,1.472) |  |
| Above high school |  | 1.335(1.145,1.556) |  |  | 1.295(1.123,1.492) |  |  | 1.350(1.192,1.528) |  |
| Not recorded |  | 0(0,0) |  |  | 0(0,0) |  |  | 0(0,0) |  |
|  |  |  |  |  |  |  |  |  |  |
| Ages 20-39 |  | 1.029(0.793,1.334) | <0.001 |  | 0.999(0.798,1.251) | 0.03 |  | 1.000(0.817,1.224) | <0.001 |
| Ages 40-59 |  | 1.217(1.015,1.459) |  |  | 1.308(1.106,1.547) |  |  | 1.365(1.180,1.579) |  |
| Ages 60+ |  | 1.418(1.222,1.646) |  |  | 1.468(1.266,1.702) |  |  | 1.440(1.268,1.634) |  |

OR Odds Ratio, CI Confidence Interval

ORs were adjusted for gender, age, race,education ,smoking,alcohol,diabetes, hyperlipidemia, pulse rate,body mass index, alanine transaminase, aspartate transaminase, total cholesterol, triglyceride, low density lipoprotein cholesterol, high density lipoprotein cholesterol, glucose, glycated hemoglobin, serum uric acid,serum creatinine and C-reactive protein.
