# Supplementary material for: The association between systemic inflammation markers and the prevalence of hypertension
Source: BMC Cardiovasc Disord. 2023 Dec 14;23:615. doi: 10.1186/s12872-023-03661-6 (PMC10720087; doi:10.1186/s12872-023-03661-6)
Supplement: Supplementary file 3 — Additional file 3: Supplementary Table 3. Effect of standardized systemic inflammation markers on hypertension: adjusted odds ratios from segmented logistic regression analysis. [file 12872_2023_3661_MOESM3_ESM.docx]

| Supplementary Table 3 Effect of standardized systemic inflammation markers on hypertension: adjusted odds ratios from segmented logistic regression analysis | | | |
| --- | --- | --- | --- |
|  | OR per SD | 95% CI | p-value |
|  |  |  |  |
| LogSII (< 2.54) | 0.92 | 0.89, 0.96 | <0.001 |
| LogSII (≥ 2.54) | 1.09 | 1.07, 1.12 | <0.001 |
|  |  |  |  |
| LogSIRI (< -0.05) | 0.99 | 0.96, 1.03 | 0.69 |
| LogSIRI (≥ -0.05) | 1.16 | 1.13, 1.19 | <0.001 |
|  |  |  |  |
| LogAISI (< 1.11) | 0.92 | 0.22, 5.21 | 0.91 |
| LogAISI (≥ 1.11) | 1.11 | 1.09, 1.13 | <0.001 |

OR Odds Ratio, CI Confidence Interval

ORs were adjusted for gender, age, race,education ,smoking,alcohol,diabetes, hyperlipidemia, pulse rate,body mass index, alanine transaminase, aspartate transaminase, total cholesterol, triglyceride, low density lipoprotein cholesterol, high density lipoprotein cholesterol, glucose, glycated hemoglobin, serum uric acid,serum creatinine and C-reactive protein.
