# Supplementary material for: The association between systemic inflammation markers and the prevalence of hypertension
Source: BMC Cardiovasc Disord. 2023 Dec 14;23:615. doi: 10.1186/s12872-023-03661-6 (PMC10720087; doi:10.1186/s12872-023-03661-6)
Supplement: Supplementary file 4 — Additional file 4: Supplementary Table 4. Spearman correlation analysis between baseline variables and blood pressure. [file 12872_2023_3661_MOESM4_ESM.docx]

| Supplementary Table 4 Spearman correlation analysis between baseline variables and blood pressure | | | | | | | | | | | | | | | | | | | | | |
| --- | --- | --- | --- | --- | --- | --- | --- | --- | --- | --- | --- | --- | --- | --- | --- | --- | --- | --- | --- | --- | --- |
|  |  | LogSII | LogSIRI | LogAISI | Age | BMI | Pulse rate | ALT | AST | TG | TC | LDL-C | HDL-C | HbA1c | GLU | Serum uric acid | Serum creatinine | CRP | SBP | DBP | MAP |
| LogSII | r | 1 | 0.735 | 0.860 | 0.001 | 0.046 | 0.201 | -0.089 | -0.121 | 0.075 | 0.048 | 0.022 | 0.010 | -0.036 | 0.008 | -0.030 | -0.078 | 0.140 | -0.005 | -0.051 | -0.036 |
|  | p | . | 0 | 0 | 0.914 | 0 | 0 | 0 | 0 | 0 | 0 | 0 | 0.035 | 0 | 0.12 | 0 | 0 | 0 | 0.268 | 0 | 0 |
| LogSIRI | r | 0.735 | 1 | 0.912 | 0.098 | 0.049 | 0.142 | -0.01 | -0.024 | 0.121 | -0.032 | -0.062 | -0.067 | 0.021 | 0.038 | 0.083 | 0.071 | 0.182 | 0.059 | -0.076 | -0.02 |
|  | p | 0 | . | 0 | 0 | 0 | 0 | 0.035 | 0 | 0 | 0 | 0 | 0 | 0 | 0 | 0 | 0 | 0 | 0 | 0 | 0 |
| LogAISI | r | 0.86 | 0.912 | 1 | 0.026 | 0.072 | 0.196 | -0.03 | -0.063 | 0.131 | 0.026 | -0.005 | -0.054 | 0.011 | 0.005 | 0.04 | -0.012 | 0.183 | 0.033 | -0.05 | -0.017 |
|  | p | 0 | 0 | . | 0 | 0 | 0 | 0 | 0 | 0 | 0 | 0.35 | 0 | 0.02 | 0.275 | 0 | 0.013 | 0 | 0 | 0 | 0 |
| Age | r | 0.001 | 0.098 | 0.026 | 1 | 0.068 | -0.153 | -0.045 | 0.094 | 0.145 | 0.111 | 0.065 | 0.043 | 0.467 | 0.369 | 0.161 | 0.261 | 0.067 | 0.487 | 0 | 0.248 |
|  | p | 0.914 | 0 | 0 | . | 0 | 0 | 0 | 0 | 0 | 0 | 0 | 0 | 0 | 0 | 0 | 0 | 0 | 0 | 0.973 | 0 |
| BMI | r | 0.046 | 0.049 | 0.072 | 0.068 | 1 | 0.104 | 0.193 | 0.023 | 0.277 | 0.046 | 0.071 | -0.293 | 0.28 | 0.239 | 0.257 | 0.024 | 0.284 | 0.16 | 0.119 | 0.155 |
|  | p | 0 | 0 | 0 | 0 | . | 0 | 0 | 0 | 0 | 0 | 0 | 0 | 0 | 0 | 0 | 0 | 0 | 0 | 0 | 0 |
| Pulse rate | r | 0.201 | 0.142 | 0.196 | -0.153 | 0.104 | 1 | 0.001 | -0.042 | 0.131 | 0.066 | 0.015 | -0.029 | -0.004 | 0.05 | -0.058 | -0.151 | 0.14 | -0.067 | 0.089 | 0.017 |
|  | p | 0 | 0 | 0 | 0 | 0 | . | 0.766 | 0 | 0 | 0 | 0.002 | 0 | 0.377 | 0 | 0 | 0 | 0 | 0 | 0 | 0 |
| ALT | r | -0.089 | -0.01 | -0.03 | -0.045 | 0.193 | 0.001 | 1 | 0.72 | 0.225 | 0.084 | 0.062 | -0.238 | 0.096 | 0.133 | 0.28 | 0.122 | -0.021 | 0.088 | 0.179 | 0.159 |
|  | p | 0 | 0.035 | 0 | 0 | 0 | 0.766 | . | 0 | 0 | 0 | 0 | 0 | 0 | 0 | 0 | 0 | 0 | 0 | 0 | 0 |
| AST | r | -0.121 | -0.024 | -0.063 | 0.094 | 0.023 | -0.042 | 0.72 | 1 | 0.121 | 0.06 | 0.003 | -0.057 | 0.072 | 0.089 | 0.243 | 0.182 | -0.047 | 0.126 | 0.105 | 0.129 |
|  | p | 0 | 0 | 0 | 0 | 0 | 0 | 0 | . | 0 | 0 | 0.485 | 0 | 0 | 0 | 0 | 0 | 0 | 0 | 0 | 0 |
| TG | r | 0.075 | 0.121 | 0.131 | 0.145 | 0.277 | 0.131 | 0.225 | 0.121 | 1 | 0.335 | 0.114 | -0.478 | 0.233 | 0.231 | 0.228 | 0.075 | 0.128 | 0.165 | 0.103 | 0.151 |
|  | p | 0 | 0 | 0 | 0 | 0 | 0 | 0 | 0 | . | 0 | 0 | 0 | 0 | 0 | 0 | 0 | 0 | 0 | 0 | 0 |
| TC | r | 0.048 | -0.032 | 0.026 | 0.111 | 0.046 | 0.066 | 0.084 | 0.06 | 0.335 | 1 | 0.851 | 0.158 | 0.054 | 0.018 | 0.037 | -0.065 | -0.006 | 0.121 | 0.158 | 0.169 |
|  | p | 0 | 0 | 0 | 0 | 0 | 0 | 0 | 0 | 0 | . | 0 | 0 | 0 | 0 | 0 | 0 | 0.241 | 0 | 0 | 0 |
| LDL-C | r | 0.022 | -0.062 | -0.005 | 0.065 | 0.071 | 0.015 | 0.062 | 0.003 | 0.114 | 0.851 | 1 | 0.01 | 0.034 | -0.01 | 0.065 | -0.035 | -0.006 | 0.083 | 0.149 | 0.145 |
|  | p | 0 | 0 | 0.35 | 0 | 0 | 0.002 | 0 | 0.485 | 0 | 0 | . | 0.034 | 0 | 0.041 | 0 | 0 | 0.182 | 0 | 0 | 0 |
| HDL-C | r | 0.01 | -0.067 | -0.054 | 0.043 | -0.293 | -0.029 | -0.238 | -0.057 | -0.478 | 0.158 | 0.01 | 1 | -0.169 | -0.19 | -0.292 | -0.17 | -0.104 | -0.042 | -0.074 | -0.067 |
|  | p | 0.035 | 0 | 0 | 0 | 0 | 0 | 0 | 0 | 0 | 0 | 0.034 | . | 0 | 0 | 0 | 0 | 0 | 0 | 0 | 0 |
| HbA1c | r | -0.036 | 0.021 | 0.011 | 0.467 | 0.28 | -0.004 | 0.096 | 0.072 | 0.233 | 0.054 | 0.034 | -0.169 | 1 | 0.526 | 0.174 | 0.157 | 0.208 | 0.306 | 0.047 | 0.182 |
|  | p | 0 | 0 | 0.02 | 0 | 0 | 0.377 | 0 | 0 | 0 | 0 | 0 | 0 | . | 0 | 0 | 0 | 0 | 0 | 0 | 0 |
| GLU | r | 0.008 | 0.038 | 0.005 | 0.369 | 0.239 | 0.05 | 0.133 | 0.089 | 0.231 | 0.018 | -0.01 | -0.19 | 0.526 | 1 | 0.186 | 0.139 | 0.139 | 0.27 | 0.035 | 0.154 |
|  | p | 0.12 | 0 | 0.275 | 0 | 0 | 0 | 0 | 0 | 0 | 0 | 0.041 | 0 | 0 | . | 0 | 0 | 0 | 0 | 0 | 0 |
| Serum uric acid | r | -0.03 | 0.083 | 0.04 | 0.161 | 0.257 | -0.058 | 0.28 | 0.243 | 0.228 | 0.037 | 0.065 | -0.292 | 0.174 | 0.186 | 1 | 0.514 | 0.078 | 0.212 | 0.121 | 0.181 |
|  | p | 0 | 0 | 0 | 0 | 0 | 0 | 0 | 0 | 0 | 0 | 0 | 0 | 0 | 0 | . | 0 | 0 | 0 | 0 | 0 |
| Serum creatinine | r | -0.078 | 0.071 | -0.012 | 0.261 | 0.024 | -0.151 | 0.122 | 0.182 | 0.075 | -0.065 | -0.035 | -0.17 | 0.157 | 0.139 | 0.514 | 1 | -0.036 | 0.191 | 0.053 | 0.126 |
|  | p | 0 | 0 | 0.013 | 0 | 0 | 0 | 0 | 0 | 0 | 0 | 0 | 0 | 0 | 0 | 0 | . | 0 | 0 | 0 | 0 |
| CRP | r | 0.14 | 0.182 | 0.183 | 0.067 | 0.284 | 0.14 | -0.021 | -0.047 | 0.128 | -0.006 | -0.006 | -0.104 | 0.208 | 0.139 | 0.078 | -0.036 | 1 | 0.08 | 0.001 | 0.038 |
|  | p | 0 | 0 | 0 | 0 | 0 | 0 | 0 | 0 | 0 | 0.241 | 0.182 | 0 | 0 | 0 | 0 | 0 | . | 0 | 0.902 | 0 |
| SBP | r | -0.005 | 0.059 | 0.033 | 0.487 | 0.16 | -0.067 | 0.088 | 0.126 | 0.165 | 0.121 | 0.083 | -0.042 | 0.306 | 0.27 | 0.212 | 0.191 | 0.08 | 1 | 0.37 | 0.746 |
|  | p | 0.268 | 0 | 0 | 0 | 0 | 0 | 0 | 0 | 0 | 0 | 0 | 0 | 0 | 0 | 0 | 0 | 0 | . | 0 | 0 |
| DBP | r | -0.051 | -0.076 | -0.05 | 0 | 0.119 | 0.089 | 0.179 | 0.105 | 0.103 | 0.158 | 0.149 | -0.074 | 0.047 | 0.035 | 0.121 | 0.053 | 0.001 | 0.37 | 1 | 0.866 |
|  | p | 0 | 0 | 0 | 0.973 | 0 | 0 | 0 | 0 | 0 | 0 | 0 | 0 | 0 | 0 | 0 | 0 | 0.902 | 0 | . | 0 |
| MAP | r | -0.036 | -0.02 | -0.017 | 0.248 | 0.155 | 0.017 | 0.159 | 0.129 | 0.151 | 0.169 | 0.145 | -0.067 | 0.182 | 0.154 | 0.181 | 0.126 | 0.038 | 0.746 | 0.866 | 1 |
|  | p | 0 | 0 | 0 | 0 | 0 | 0 | 0 | 0 | 0 | 0 | 0 | 0 | 0 | 0 | 0 | 0 | 0 | 0 | 0 | . |

ALT alanine transaminase, AST aspartate transaminase, TC total cholesterol, TG triglyceride, LDL-C low density lipoprotein cholesterol, HDL-C high density lipoprotein cholesterol, GLU glucose, HbA1c glycated hemoglobin, CRP C-reactive protein, SBP systolic blood pressure,DBP diastolic blood pressure,MAP mean arterial pressure
